# Gender-Differentiated Parenting Revisited: Meta-Analysis Reveals Very Few Differences in Parental Control of Boys and Girls

**DOI:** 10.1371/journal.pone.0159193

**Published:** 2016-07-14

**Authors:** Joyce J. Endendijk, Marleen G. Groeneveld, Marian J. Bakermans-Kranenburg, Judi Mesman

**Affiliations:** Centre for Child and Family Studies, Leiden University, Leiden, the Netherlands; Philipps University Marburg, GERMANY

## Abstract

Although various theories describe mechanisms leading to differential parenting of boys and girls, there is no consensus about the extent to which parents *do* treat their sons and daughters differently. The last meta-analyses on the subject were conducted more than fifteen years ago, and changes in gender-specific child rearing in the past decade are quite plausible. In the current set of meta-analyses, based on 126 observational studies (15,034 families), we examined mothers’ and fathers’ differential use of autonomy-supportive and controlling strategies with boys and girls, and the role of moderators related to the decade in which the study was conducted, the observational context, and sample characteristics. Databases of Web of Science, ERIC, PsychInfo, Online Contents, Picarta, and Proquest were searched for studies examining differences in *observed* parental control of boys and girls between the ages of 0 and 18 years. Few differences were found in parents’ use of control with boys and girls. Parents were slightly more controlling with boys than with girls, but the effect size was negligible (*d* = 0.08). The effect was larger, but still small, in normative groups and in samples with younger children. No overall effect for gender-differentiated autonomy-supportive strategies was found (d = 0.03). A significant effect of time emerged: studies published in the 1970s and 1980s reported more autonomy-supportive strategies with boys than toward girls, but from 1990 onwards parents showed somewhat more autonomy-supportive strategies with girls than toward boys. Taking into account parents’ gender stereotypes might uncover subgroups of families where gender-differentiated control is salient, but based on our systematic review of the currently available large data base we conclude that in general the differences between parenting of boys versus girls are minimal.

## Introduction

One of the mechanisms proposed to explain gender differences in children’s behavior is that parents treat boys and girls differently [1]. Several theoretical models suggest mechanisms that are consistent with the differential treatment of boys and girls, including biosocial theory [2], [3], and gender schema theories [4], [5]. However, to date there is no consensus in the literature about the extent to which parents *do* treat their sons and daughters differently, in which areas of parenting this mostly occurs, and whether fathers and mothers differ in the extent of gender differentiation [6], [7], [8]. We conducted a series of meta-analyses to examine whether parents use different control strategies with boys than with girls. We focused on *observed* parental control, to minimize social desirable responding by parents and because differential parenting occurs mostly at an unconscious level and is therefore more likely to be captured using observation methods than with self-report measures [9].

### Gender-Differentiated Control: Theoretical Perspectives

#### Self-determination theory

Parental control strategies can be defined as any strategy that a parent uses to alter, change, or influence their child’s behavior, thoughts, or feelings [10], [11]. Self-determination theory [12] provides a framework for different types of parental control that promote optimal or less optimal child development. Central to this theory is the distinction between behaviors that a person willingly endorses (i.e., autonomously regulated behavior) and behaviors that are enacted because of pressure from, for example, the social environment (i.e., controlled behavior). Self-determination theory assumes that two types of parental control play an important role in children’s development of autonomous or controlled regulation of behavior [13], [14], i.e., autonomy-supportive and controlling strategies [15].

Autonomy-supportive strategies provide the child with an appropriate amount of control, a desired amount of choice, acknowledge the child’s perspectives, and provide the child with meaningful rationales when choice is constrained [14]. These strategies are thought to foster autonomous regulation and child well-being, because they adhere to children’s basic needs for competence, relatedness, and autonomy [12]. Autonomy-supportive strategies are conceptually similar to the construct of parental sensitivity as formulated within attachment theory, as sensitivity is also concerned with child-centered responding and promoting autonomy through support [16], [17]. Examples of autonomy-supporting strategies are induction (i.e., providing explanations for commands and prohibitions), empathy for the child (“I know this is difficult for you”), approval, support, and encouragement (see [11], [18]). Meta-analyses have shown that maternal and paternal autonomy-supportive strategies tend to be associated with lower levels of child disruptive behaviors such as oppositional, aggressive, and hyperactive behaviors [19], [20], [21]. Furthermore, a previous study has also shown that an intervention to promote mothers’ use of autonomy-supportive strategies (i.e., sensitive discipline) was effective in decreasing children’s disruptive (i.e., overactive) behavior [22].

Controlling strategies undermine the child’s ability for autonomous regulation, and press the child to think, behave, or feel in particular ways [14], [15]. These strategies are thought to foster controlled regulation and behavioral maladjustment, because they do not support children’s basic needs for competence, relatedness, and autonomy [12]. Controlling strategies are conceptually similar to the parenting practices described within coercion theory [23]. Coercive parenting also refers to strategies that force rather than motivate a child to comply without fostering the child’s autonomy. There are two ways in which parents can be controlling [15], that is, via internal and external pressure. External pressure refers to harsh, explicit, or tangible control, such as spanking, hitting, grabbing with force, or forcefully taking the child out of the situation (i.e., harsh discipline/power assertion; [24]). Internal pressure refers to parental behaviors that intrude upon the child’s psychological world (i.e., thoughts and feelings) as a pressure to comply, and includes manipulative parenting techniques, such as guilt induction, shaming, criticism, invalidation of the child’s feelings, and love withdrawal (i.e., psychological control; [10]). There is ample empirical evidence that maternal and paternal controlling behavior in general is related to an increase in disruptive behavior in children of different ages (see meta-analyses [19], [25]). Moreover, both mothers’ and fathers’ use of psychological control is associated with internalizing problems in children and adolescents [10], [15], [26], [27], [28], and with girls’ relational aggression in middle childhood [29]. Mothers’ and fathers’ harsh physical discipline is more often associated with externalizing problems in children [30] and adolescents [31].

Self-determination theory cannot be applied to the study of gender-differentiated parental control as one of its fundamental assumptions is the universality of its psychological constructs across gender. Therefore, in the current meta-analysis the hypotheses with regard to the direction of gender-differentiated control (i.e., used more with boys or girls) were guided by theoretical frameworks addressing socialization and gender development, including biosocial theory [2], [3], and gender schema theories (e.g., [4], [5]).

#### Biosocial theory

Biosocial theory of sex differences provides rationales for differential control of boys and girls [2], [3]. According to this theory, gender differences in social behavior arise from societies’ division in gender roles, and particularly on the female role of homemaker and the male role of economic provider. This division is still visible in present-day societies; mothers are more likely to be the primary caregivers of young children [32], [33], females are overrepresented in educational and nurturing occupations, and males are overrepresented in occupations that are associated with power, physical strength, status, and agentic personality characteristics (i.e., management, engineering) [34].

Biosocial theory proposes the following cycle in which gender roles and the characteristics associated with these roles lead to beliefs and expectancies about the different nature and behavior of men and women (i.e., gender stereotypes), which will lead to differential treatment of men and women, and boys and girls [3]. Mothers and fathers are expected to use different control strategies with boys than with girls in accordance with the gender roles defined in their society. Parental control of girls would be characterized by kindness, consideration of others’ perspectives, empathy, and interpersonal closeness (e.g., using autonomy-supportive strategies), whereas parental control of boys would be characterized by power, assertiveness, aggressiveness, and dominance (e.g., using controlling strategies). The link between gender roles and the differential treatment of boys and girls by parents is reflected, for example, in the finding that aggressiveness is promoted in boys, and not in girls, through harsh parenting practices in societies at war [35]. Since women are less accepting than men of social hierarchies that subordinate women [36], mothers may be less likely than fathers to socialize their children into societies’ gender roles using gender-differentiated parenting practices.

#### Gender schema theories

It seems unlikely that all parents in a given society would use gender-differentiated control strategies in accordance with the gender roles of that society. According to gender schema theories [4] parents’ gender-differentiated use of controlling and autonomy supportive strategies is likely to be influenced by parents’ gender-role stereotypes. When parents have traditional attitudes about gender roles, they are more likely to show gender-differentiated parenting that reinforces gender-role consistent behavior (e.g., more harsh or physical control of boys than girls, more gentle control and guidance of girls than of boys). When parents have counter-stereotypical ideas about the roles of males and females (i.e., female as economic provider, male as caretaker), they might be more likely to show gender-differentiated parenting that reinforces behavior that is inconsistent with gender roles (e.g., more gentle control and guidance of boys than of girls, more harsh or physical control of girls than of boys).

### Gender-Differentiated Parental Control: Previous Findings

There is some meta-analytic evidence that parents use different control strategies with boys and girls, and that the extent to which this happens differs for fathers and mothers. For example, Lytton and Romney [8] demonstrated in their meta-analysis that in Western countries other than North America, parents use more physical punishment with boys than with girls. They also found some evidence for fathers to differentiate more between boys and girls than mothers. In their meta-analysis, Leaper and colleagues [7] found that mothers used more supportive speech with daughters than with sons, with greater effects for older than younger children. They also found a negligible effect for mothers’ use of directive speech (i.e., slightly more with girls than with boys).

Both meta-analyses are cited broadly, but they were not without limitations [7], [37]. First, both meta-analyses did not disentangle child gender effects on parenting from effects of temperament or gender-specific behavioral differences, probably because too few studies included pertinent data. Second, the Lytton and Romney meta-analysis [8] has been criticized for using categories of socialization behaviors that were too broad [37], and combining constructs that were too divergent. However, choosing a construct that is too specific harbors the risk of ending up with only a few studies on fathers, as was the problem in the Leaper, Anderson, and Sanders meta-analysis [7]. Third, both meta-analyses did not include psychological control. To our knowledge the literature on psychological control has not yet been systematically reviewed with regard to the differential use of psychological control with boys and girls.

Some recent observation studies have found similar results as the meta-analyses, with parents using more sensitive or autonomy-supportive strategies with girls than with boys (e.g., [38], [39]) and more harsh or controlling strategies with boys than with girls (e.g., [39], [40]). These findings indicate a tendency for controlling strategies (i.e., focused on dominance, negativity, and power) to be used preferably with boys, and autonomy-supportive strategies (i.e., focused on warmth, affiliation, and interpersonal closeness) to be used more with girls.

However, there is also a large number of recent studies that does not find evidence for parents’ gender-differentiated use of control (e.g.,[41], [42], [43], [44]). Additionally, some studies even show that parents use more autonomy-supportive strategies with boys than with girls (e.g., [45], [46]), and are more controlling of girls than of boys (e.g., [46], [47]). The evidence with regard to parents’ differential use of psychological control is especially inconsistent, indicating that parental psychological control is higher among boys than girls [29], [48], or that there are no gender differences in the use of psychological control [49].

### Factors Related to Gender-Differentiated Parenting

#### Observational context

An important question with regard to the magnitude of gender differences in parental control is whether this difference is context-specific. In the meta-analysis by Leaper et all [7] less structured and more naturalistic situations and activities yielded the greatest gender differences. Leaper and colleagues suggest that this might be due to the fact that in highly structured situations the demand characteristics of the task will lead to a smaller range of possible behaviors, which minimizes naturally occurring differences in parenting and child behavior. In the current meta-analysis, we expected the naturalistic context–in which parent and child are allowed to behave as they would normally do–to yield the greatest gender differences because it is the least structured situation, followed by free play, followed by more structured tasks such as problem-solving tasks, and discipline tasks (e.g., “Clean up”, “Don’t touch”, delay of gratification)[50]. The distinction between these four types of activities is quite common in studies on observed parenting practices [50]. In fact, they reflect a continuum of structured to non-structured activities.

#### Child behavior

Differential control of boys and girls may not, or not only, result from parental attitudes about how to treat boys versus girls, but as a reaction to pre-existing gender differences in child behavior. Large longitudinal studies with ethnically and socioeconomically diverse samples provide ample evidence for the bidirectional association between parental controlling or autonomy-supportive strategies on the one hand and child disruptive behaviors at the other hand (see [51], [52], [53]). Similarly, large population-based longitudinal twin studies from the US and UK have shown that cooperative and/or prosocial children (aged 2–12 years old) are more likely to elicit positive reactions from their mothers and fathers, whereas children with tendencies toward disruptive behavior elicit negative reactions from their mothers and fathers (evocative rGE, [51], [54], [55]). Given this evidence and the fact that boys have been found to show more disruptive behavior problems than girls during childhood and adolescence [56], [57], [58], [59], and because boys have shown more genetic liability for disruptive behavior problems than girls [60], [61]), they may also be more likely to elicit controlling behavior from their parents.

There is at least one study showing that it is not only a gender difference in child behavior that elicits the different treatment of boys and girls. In this 10-year longitudinal population-based study of approximately 1,000 US children between the ages of 1 and 20 years it was found that mothers and fathers were harsher with boys than with girls [62]. Boys and girls in this study did not differ in terms of temperament, so the harsher treatment of boys was not because they were more difficult to begin with. As a response to this harsh treatment, especially by mothers, boys appeared to become more difficult and noncompliant. However, it should be noted that this is a single study, relying on questionnaires and interviews, without observational data. Thus, potential effects of child temperament or behavior on gender-differentiated parenting cannot be ruled out conclusively.

In the current meta-analysis we tried to take the child’s behavior during the task into account (e.g., using proportion scores, or including child behavior as a covariate in the analyses), to disentangle differences in parental control toward boys and girls from differences in behavior of boys and girls. We expected effect sizes to be larger in studies that did not control for child behavior, because in these studies the child effect on gender-differentiated parenting is not controlled for. In a related vein we expected parents’ differential use of controlling or autonomy-supportive strategies to be less pronounced in clinical or at risk samples (e.g., child has some disorder, or shows high or clinical levels of problem behavior) compared to healthy samples. In these samples boys and girls show more similar levels of problem behavior, and are thus unlikely to elicit differential reactions by their parents based on their behavior. Alternatively, the similar level of child problems in boys and girls in these families may be the consequence of parents’ similar use of controlling and autonomy-supportive practices with boys and girls that may have caused the problem behaviors in the first place.

#### Child age

Variation in effect sizes for gender differences in parental control may also be related to developmental level. The evidence with regard to developmental level is, however, inconclusive. Biosocial theory does not explicitly incorporate child age effects [2], [3]. However, pressures to conform to gender roles increase with child age, and the pressure to conform might be highest in adolescence [63]. Gender-specific parenting may increase as children get older in order to prepare children for the greater pressures toward gender role conformity [64]. There is also meta-analytic evidence convergent with these propositions; Leaper and colleagues [7] found that gender differences in mothers’ directive speech were greater with older children than with younger children. However, Lytton and Romney [8] found that gender differences actually decreased with age, specifically for disciplinary strictness. With regard to parental control, one might argue that gender differences in parental control decrease with child age, because parental control generally decreases over time due to increases in children’s self-control [65]. These generally lower levels of parental control with older children may reduce the statistical power to detect differential treatment of boys and girls, leading to smaller effect sizes. Therefore, we tested two competing hypotheses; 1) parents’ gender-differentiated control increases with child age; 2) parents’ gender-differentiated control decreases with child age.

#### Socioeconomic status (SES) and culture

Parents’ SES and cultural backgrounds may also be a moderator of the differential control of boys and girls. There is ample evidence that higher SES (i.e., education, salary) is associated with less traditional views on gender roles [66], [67], [68]. Similarly, there is evidence that lower-SES families show more gender-differentiated parenting than middle-class families [69]. This is indeed what would be expected in light of biosocial theory [2], [3], because the more traditional views about gender roles in lower-SES families would lead to a bigger differentiation between boys and girls. In the current meta-analysis, we expected the differential control of boys and girls to be greater in lower-SES families compared to middle-class families.

There may also be cultural variation in the way parents treat boys and girls. From the perspective of biosocial theory [2], [3], one might argue that in cultures with big differences in the gender roles of men and women (i.e., big gender gap), parents will differentiate more between their sons and daughters to prepare them for adult life in a culture with big differences in gender roles. Data on the gender gap (gender differences in health, life expectancy, access to education, economic participation, salaries, job type, and political engagement) showed that Scandinavian and Western European countries generally have the lowest gender gap in the world [70], and that North-American countries have a somewhat bigger gender gap. Latin-American and Asian societies have intermediate levels of gender inequality. The largest gender inequality can be found in Middle-East and North-African societies. Thus, with regard to the ethnicity of the sample, we expected gender differences in control of boys and girls to be smaller in cultures where there are small differences in the roles of men and women (e.g., Western vs Eastern countries).

#### Publication year

In recent decades the division of gender roles has become less strict in most modern Western societies [71], [72], which according to biosocial theory would lead to more egalitarian attitudes about gender, and consequently less differentiation between boys and girls [2], [3]. Moreover, gender equality has increased in most Western societies over the decades [73]. Therefore, we expected that effect sizes would be smaller in recent studies compared to older studies.

#### Other moderators

We also examined some moderators in an explorative way, because they were also examined or proposed in previous meta-analyses [7], [8]; observation length, home versus lab setting, verbal versus nonverbal behavior, gender of the coders of parenting behavior, gender of the first author, percentage of male authors, and publication outlet. No clear predictions could be made for these moderators.

### The Current Study

The current meta-analysis was conducted to determine the extent to which parents control their sons and daughters differently. We tested the following hypotheses based on biosocial theory and previous meta-analyses: (a) mothers and fathers use more controlling strategies, including psychological control and harsh physical discipline, with their sons than with their daughters [2], [3], [8], [48]; (b) mothers and fathers use more autonomy-supportive strategies with their daughters than with their sons [2], [3], [7]; (c) fathers’ controlling and autonomy-supportive strategies are more gender-differentiated than mothers’ controlling and autonomy-supportive strategies [2], [3], [8]. A conceptual analysis with expert raters was used to classify parental control variables as controlling and autonomy-supportive.

Aspects of the current meta-analyses that extend previous meta-analytic work include: 1) a focus on parental control as a specific construct to examine gender-differentiated parenting, including psychological control and harsh physical control, 2) a comparison between mothers’ and fathers’ parental control, 3) an examination of the effect of procedural moderators, 4) a comparison of studies that control and do not control for child behavior, thus addressing alternative explanations for gender-differentiated parental control, and 5) the inclusion of studies that have been conducted during the past two decades.

## Methods

### Literature Search

The PRISMA guidelines were used for conducting and reporting the current meta-analysis [74] (see S1 Text). There is no review-protocol for the current meta-analysis. Three search methods were used to identify eligible studies published up until June 1st, 2015. First, the electronic databases of Web of Science (WOS), ERIC, PsychInfo, Online Contents, Picarta, and Proquest Dissertations and Theses were searched for empirical, peer-reviewed articles using the keywords for parental control in observational settings (see S2 Text). For WOS, additional restrictions were used based on WOS categories. These restrictions are listed in S1 Table.

Studies were included if they: a) examined differences in parental control of boys and girls between the ages of 0 and 18 years; b) used observations of parental control (e.g., free play, problem solving, discipline setting, naturalistic). Control was defined as “strategies parents use to alter the child’s behavior”. Studies were excluded if parental control was assessed in relation to gender socialization (e.g., parental control of sex-typed play), as this was considered to be a different socialization area. There were no restrictions with regard to the language of the paper, as long as an English abstract was available for screening purposes. During the full-text screening phase, papers that were written in languages other than English (one Turkish, one Chinese, three Spanish, one French, and two German) were translated by native speakers. Of the included publications, one was published in German and one in Spanish.

**Fig 1:**
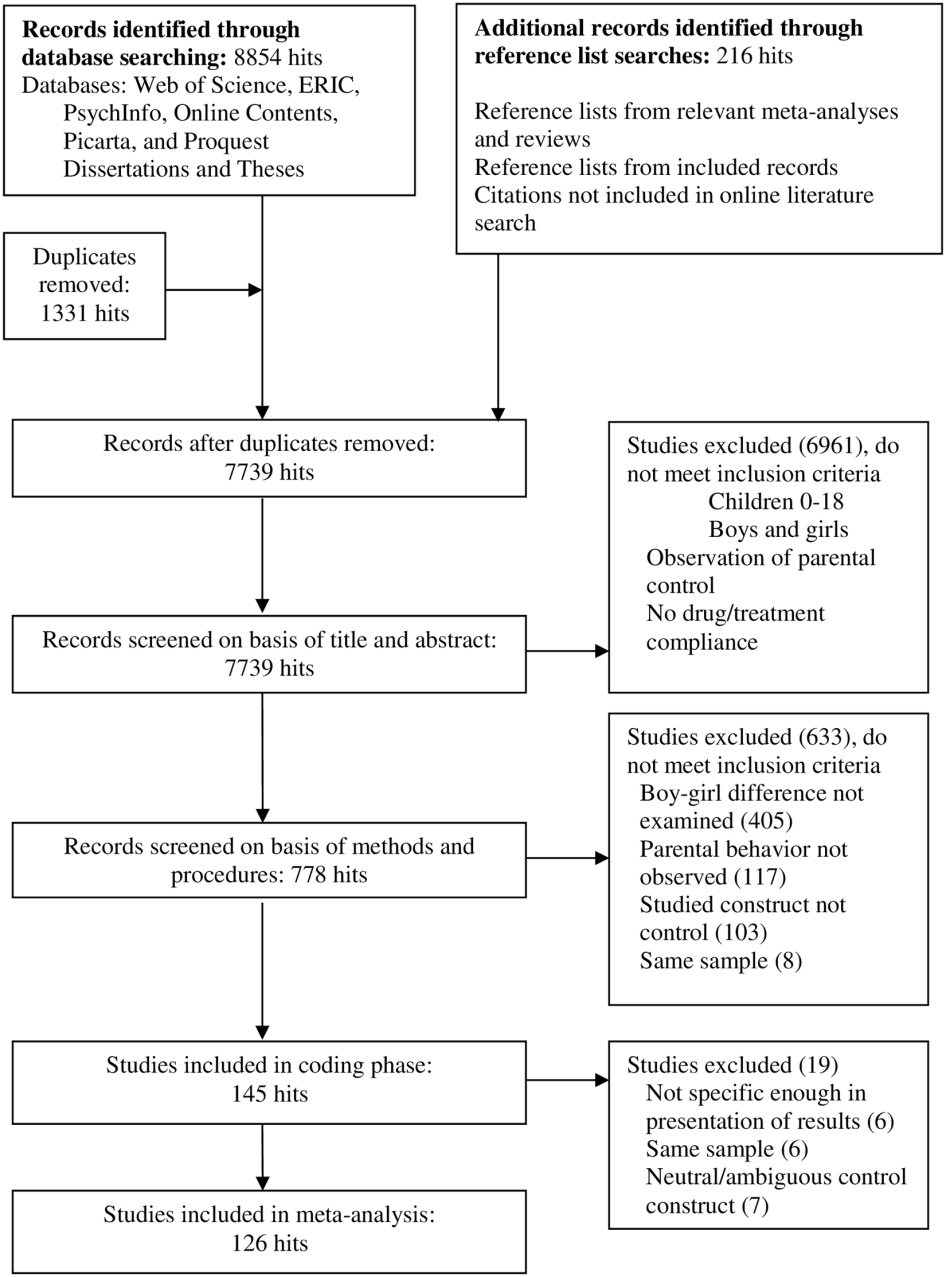
Flow-Chart of Literature Search Process.

First, we checked whether the search terms yielded all discipline-related articles included in the Lytton and Romney [8] meta-analysis. This was indeed the case. Second, we searched the reference lists of relevant reviews and meta-analyses on parental control [7], [20], [25], [75]. Third, the reference lists of the articles and dissertations that met our inclusion criteria were also searched for eligible studies. We applied a very broad strategy with this reference search, including all articles that mentioned any of our search in the title terms, or one of the following more general constructs: parenting, socialization, parent-child interaction/speech, parental behavior/behaviour. The database search and reference list search together yielded 7739 hits. Fig 1 depicts the flow chart of the literature search.

Agreement between the first and second authors on the inclusion of studies was determined on a random subset of 100 studies, oversampling included studies. Studies were first screened only on the basis of their abstracts, followed by a full-text screening of the selected studies. Agreement was satisfactory for both the abstract screening (agreement 92%) and the full-text screening (agreement 100%). Disagreements between the authors were resolved by discussion until consensus was achieved. After the reliability assessment, the first author screened the remainder of the articles, but consulted the second author in cases of doubt.

To ascertain the independence of samples in the meta-analysis, several precautions were taken. First, for studies conducted on the same sample, the publication with the maximum or most relevant information was included. Second, when a publication separately reported gender-differentiated control for more than one sample (e.g., different age groups, different ethnicities), these sub-samples were treated as independent samples, but only if the sub-sample was relevant to one of the moderators of the current study (e.g., age, normative sample, observation setting). For other sub-samples (e.g., long divorced vs. recently divorced) a combined effect size was calculated. Third, when a publication reported different outcomes on the same sample, they were averaged if they concerned the same type of parental control (e.g., praise and guidance averaged for autonomy-supportive strategies). If they reported outcomes on different observation settings (e.g., free play, teaching task, discipline task) they were averaged for the overall meta-analysis, but for the analyses with task setting as moderator one of the settings was randomly selected. This procedure yielded 126 publications with data from 146 independent samples encompassing a total of 15,034 families. The studies that were included in the meta-analyses are presented in Table 1.

**Table 1:** Studies Included in the Meta-Analysis.

Study	Parent^a^	Control type^b^	Sample size		% ♀	Age (in years)	Ethnicity^c^	Task^d^	Sample normative	SES^e^	Setting^f^	Only verbal	Other moderators^g^					
													1	2	3	4	5	6
Ahl et al. 2013 [45]	M	+	8 ♀	8 ♂	50	1.0	-	F	Yes	4	H	No	28	-	1	1	50	1
Barkley 1989 [76]	M	+, -	20 ♀	20 ♂	50	6.0	-	F, T, M	No	4	L	No	20	-	1	1	100	1
Barnett et al. 1998 [77]	M	-	38 ♀	31 ♂	55	4.6	AA	F	Yes	1	L	No	7	-	2	1	67	1
Baumrind 1971 [78]	M, F	+, -	69 ♀	80 ♂	46	4.2	-	N	Yes	4	H	No	-	-	1	2	0	1
Befera et al. 1985 [79]	M	+, -	30♀	30 ♂	50	8.6	-	F, T, M	Yes, No	4	L	No	10	-	1	2	50	1
Belden et al. 2007 [80]	M	+, -	133 ♀	144 ♂	48	4.0	-	D	No	3	L	No	8	-	1	1	33	1
Bellinger et al. 1982 [81]	M, F	-	5 ♀	5 ♂	50	3.9	-	T	Yes	3	L	Yes	30	-	1	1	50	1
Bernstein et al. 2005 [82]	M	+	332 ♀	351 ♂	49	4.0	Mixed	T	Yes	1	L	No	-	-	2	1	20	1
Blackwelder et al. 1986 [83]	M	+, -	12 ♀	12 ♂	50	5.9	-	T	Yes	4	L	No	-	-	2	1	100	1
Braungart-Rieker et al. 1997 [18]	M	+, -	29 ♀	28 ♂	51	2.5	Mixed	D	Yes	2	L	No	2	-	2	2	0	1
Bright et al. 1984 [84]	M, F	+, -	13 ♀	16 ♂	45	4.7	-	F	Yes	2	L	No	10	2	1	2	0	1
Brody et al. 1985 [85]	M	+, -	20 ♀	14 ♂	42	5.2	-	N	Yes	2	H	No	40	-	2	1	100	1
Brody et al. 1986 [86]	M, F	+, -	23 ♀	37 ♂	38	6.5	NAC	T	Yes	3	L	No	5	-	2	1	100	1
Brody et al. 1992 [87]	M, F	+, -	53 ♀	56 ♂	49	7.5	NAC	T	Yes	3	H	No	-	-	2	1	33	1
Bronstein 1984 [88]	M, F	+, -	24 ♀	30 ♂	43	9.0	SA	N	Yes	1	H	No	60	-	1	2	0	1
Bronstein et al. 2007 [89]	C	+, -	51 ♀	42 ♂	55	10.7	NAC	N	Yes	4	H	No	60	-	1	2	0	1
Caldera et al. 1989 [90]	M, F	+	20 ♀	20 ♂	50	1.7	-	D	Yes	-	L	Yes	24	-	1	2	0	1
Calkins et al. 1998 [91]	M	+, -	35 ♀	30 ♂	54	2.0	Mixed	T	Yes	2	L	No	11	-	2	2	0	1
Campbell et al. 1986 [92]	M	+, -	27 ♀	41 ♂	40	2.9	-	F	No	-	L	No	15	-	2	2	0	1
Campbell 1999 [93]	M	+, -, H	66 ♀	73 ♂	47	10	Mixed	T	Yes	2	L	Yes	20	-	1	2	0	2
Celano et al. 2008 [94]	M	+	29 ♀	72 ♂	29	8.6	Mixed	T	No	1	L	No	15	-	2	2	33	1
Chaplin et al., 2014 [95]	M	+, -	32 ♀	26 ♂	55	15.1	Mixed	T	Yes	3	L	No	10	-	2	2	17	1
Chen et al. 2000 [96]	M	+, -	84 ♀	82 ♂	51	2.0	C	F	Yes	4	L	No	19	-	2	2	100	1
Chen et al. 2001 [41]	M, F	+, -	40 ♀	28 ♂	59	4.2	C	T	Yes	4	H	No	30	-	2	1	50	1
Cherry et al. 1976 [97]	M	-	6 ♀	6 ♂	50	2.0	-	F	Yes	-	L	Yes	15	-	1	2	50	1
Christopoulou 1988 [98]	M	-	36 ♀	32 ♂	53	7.3	Mixed		Yes	2	L	No	10	-	2	2	0	2
Ciarrocchi 1983 [99]	M	+, -	31 ♀	27 ♂	53	5.2	-	T	Yes	3	H	No	3	-	2	1	100	2
Cipriano et al. 2010 [100]	M	+	63 ♀	63 ♂	50	2.0	Mixed	D	Yes	4	L	No	4	-	2	2	0	1
Copeland 1985 [101]	M	+, -	30 ♀	31 ♂	49	8.5	-	T	Yes	-	L	No	50	-	1	2	0	1
Coulson 2002 [102]	M, F	P	61 ♀	52 ♂	54	4.0	Mixed		Yes	4	L	No	12	-	2	2	0	2
Crockenberg et al. 1990 [103]	M	+, -	39 ♀	56 ♂	41	2.0	Mixed	N, T, M	Yes	4	H,L	No	21	-	2	2	0	1
Cyr et al. 2014 [104]	M	+, -	45 ♀	37 ♂	55	4.5	Mixed	M	Yes	1	L	No	25	-	2	2	50	1
Deater-Deckard 2000 [105]	M	+, -	120 ♀	120 ♂	50	3.6	Mixed	T	Yes	4	H	No	20	-	2	1	100	1
Dekovic et al. 1992 [106]	C	+, -	113		-	8.9	WEC	T	Yes	4	H	No	20	-	1	2	50	1
Dennis 2006 [107]	M	+, P	55 ♀	58 ♂	49	4.0	Mixed	D, F, M	Yes	4	L	No	8	-	2	2	0	1
Domenech et al. 2009 [46]	C	+, -	57 ♀	38 ♂	58	6.6	Mixed	T	Yes	1	L	No	18	3	1	2	0	1
Donovan et al. 2000 [108]	M	+, -	29 ♀	28 ♂	51	2.0	NAC	D	Yes	3	L	No	15	-	2	2	67	1
Dumas et al. 1995 [109]	M	+, -	69 ♀	57 ♂	55	4.2	Mixed	T	No	4	L	No	18	-	2	1	67	1
Eddy et al. 2001 [42]	M, F	-	201 ♀	195 ♂	51	5.0	Mixed	N	Yes	4	L	No	60	-	1	1	33	1
Eiden et al. 2001 [110]	M, F	+, -	107 ♀	108 ♂	50	1.5	Mixed	F	No	4	L	No	10	2	1	2	67	1
Eley et al. 2010 [111]	M	-	296 ♀	234 ♂	56	8.0	Mixed	T	No	4	L	No	8	-	2	2	0	1
Emmons 2001 [112]	M, F	+	49 ♀	63 ♂	41	1.6	Mixed	D	Yes	4	L	No	5	-	1	2	0	2
Fagot 1985 [113]	M, F	+, -	18 ♀	18 ♂	50	1.9	-	N	Yes	-	H	No	420	3	1	2	0	1
Fagot et al. 1993 [114]	M, F	+, -	65 ♀	72 ♂	46	1–1.5	Mixed	N	Yes	4	H	No	60	-	1	2	0	1
Fagot et al. 1996 [115]	M	+, -	46 ♀	47 ♂	49	2.5	Mixed	T	Yes	1	L	No	-	-	1	2	0	1
Falender et al. 1975 [116]	M	+, -, H	19 ♀	20 ♂	49	5.0	AA	T	Yes	1	L	No	20	-	2	2	50	1
Feldman et al. 1986 [117]	M	-	46 ♀	48 ♂	49	2.5	I	D	Yes	-	L	No	13	-	2	2	0	1
Feldman et al. 2003 [118]	M, F	+	16 ♀	16 ♂	50	2.2	I	D	Yes	2	H	No	8	-	2	2	0	1
Fisher et al. 1993 [119]	M, F	-	90 ♀	102 ♂	47	5.0	-	N	Yes	-	H	No	120	-	1	1	50	1
Frampton 2012 [120]	M	+, -	743		-	2.8	Mixed	T	Yes	4	H	No	15	-	2	2	0	2
Frankel et al. 1983 [121]	M, F	+, -	9 ♀	9 ♂	50	6.1	-	F, T, M	Yes	-	H	No	8	1	1	1	100	1
Frodi et al. 1985 [122]	M	-	17 ♀	24 ♂	41	1.0	NAC	T	Yes	4	L	No	6	-	2	2	0	1
Gaertner et al. 2008 [123]	M	+	115 ♀	141 ♂	45	1.5	Mixed	D	Yes	4	L	No	-	-	2	2	0	1
Gjerde et al. 1991 [124]	M, F	+, -	46 ♀	42 ♂	53	5.0	Mixed	T	Yes	4	L	No	-	-	1	1	67	1
Gordon 1983 [125]	M	+, -	39 ♀	35 ♂	54	3.5	Mixed	T	Yes, No	4	L	No	10	-	1	2	0	1
Gross et al. 2009 [126]	C	+, -	112 ♀	141 ♂	44	3.0	-	F, T, M	Yes	1	L	No	10	3	2	2	33	1
Gunnoe et al. 1999 [127]	M, F	+, -	217 ♀	240 ♂	49	12.9	Mixed	T	Yes	-	H	No	10	-	2	2	33	1
Gustafsson et al. 2012 [128]	M	-	338 ♀	367 ♂	48	1.3	Mixed	F	Yes	-	H	No	30	-	2	2	0	1
Henderson 2007 [129]	M	+, -	35 ♀	20 ♂	64	2.0	Mixed	D	Yes	1	H	No	5	-	1	2	0	2
Hess et al. 1984 [130]	M	-	33 ♀	34 ♂	43	4.0	NAC	T	Yes	4	L	Yes	-	-	2	1	50	1
Higgins 2008 [131]	M, F	+, -	50 ♀	50 ♂	50	2.0	Mixed	M	Yes	4	L	No	35	-	2	2	0	2
Holt 2008 [132]	M	-	53 ♀	58 ♂	48	2.0	Mixed	T	Yes	4	L	No	10	-	1	2	0	2
Huang et al. 2014 [133]	M	+, -	45 ♀	45 ♂	50	6.0	C, WEC	D	Yes	3	H	No	-	-	2	2	50	1
Huber 2012 [134]	M	-	39 ♀	41 ♂	49	0.9	SA	F	Yes	1	L	No	4	-	1	2	0	1
Hughes et al. 1999 [135]	M	+, -	138 ♀	100 ♂	58	3.6	Mixed	T	Yes	4	H	No	20	-	1	2	33	1
Inoff-Germain et al. 1988 [136]	M, F	-	30 ♀	30 ♂	50	12.3	NAC	T	Yes	2	H	No	45	-	1	2	0	1
Janssens et al. 1997 [137]	M, F	+	62 ♀	63 ♂	50	4–8	-	T	Yes	4	H	Yes	20	-	2	1	50	1
Kagan et al. 1963 [138]	M	-, P	20 ♀	30 ♂	40	4.3	-	N	Yes	4	H	No	180	-	2	1	50	1
Kalpidou et al. 1998 [139]	M	+,-, P,H	22 ♀	22 ♂	50	4.0	Mixed	D	Yes	3	L	No	27	2	2	2	33	1
Kapungu et al. 2006 [140]	M	+, -	157 ♀	117 ♂	57	11.0	AA	T	Yes	1	L	No	60	-	1	2	33	1
Kauffman 1985 [141]	M, F	-	17 ♀	23 ♂	43	5.0	-	T	Yes	4	H	Yes	5	-	1	2	0	2
Kenny-Benson et al. 2005 [142]	M	-	52 ♀	52 ♂	50	8.2	Mixed	T	Yes	3	L	No	15	-	2	2	0	1
Kerig et al. 1993 [143]	M, F	+, -	19 ♀	19 ♂	50	3.6	Mixed	F	Yes	2	L	Yes	10	2	1	2	33	1
Kochanska 1995 [144]	M	+, -,H	51 ♀	52 ♂	50	2.7	Mixed	D	Yes	4	Mix	No	80	-	2	2	0	1
Kochanska et al. 2003 [145]	M	-	53 ♀	55 ♂	49	1.2	Mixed	D	Yes	4	L	No	58	-	2	2	0	1
Kochanska et al. 2009 [40]	M, F	-	50 ♀	50 ♂	50	2.0	Mixed	D	Yes	4	L	No	45	-	2	2	25	1
Kok et al. 2012 [146]	M	+, -	214 ♀	222 ♂	49	3.1	WEC	D	Yes	4	L	No	2	-	2	2	56	1
Kuczynski 1984 [147]	M, F	+, -	32 ♀	32 ♂	50	4.0	-	T	Yes	4	L	No	9	-	1	1	100	1
LaFreniere et al. 1992 [148]	M	+, -	66 ♀	60 ♂	52	3.9	NAC	T	Yes	-	L	No	18	-	2	1	100	1
Laosa 1978 [149]	M	+, -, H	23 ♀	20 ♂	53	5.8	SA	T	Yes	4	H	No	10	2	2	1	100	1
Lengua et al. 2007 [150]	M	+, -	80		-	3.0	Mixed	T	Yes	4	L	No	-	-	2	2	0	1
Lengua et al. 2014 [151]	M	+, -	103 ♀	103 ♂	50	3.1	Mixed	M	Yes	4	L	No	24	-	2	2	0	1
Li and Lee 2013 [152]	C	+, P	150		-	7.4	Mixed	D	No	-	L	No	20	-	2	1	100	1
Lindsey et al. 2005 [153]	M	+, -	27 ♀	28 ♂	49	1.2	Mixed	T	Yes	4	H	Yes	-	-	1	1	50	1
Linver et al. 2002 [47]	M	+, -	256 ♀	237 ♂	52	2.5	Mixed	F	No	4	L	No	8	-	2	2	0	1
Liu et al. 2010 [154]	M	+, -	42 ♀	37 ♂	53	5.2	C, NAC	F	Yes	3	L	No	30	-	2	2	50	1
Lloyd 2010 [155]	M	-	13 ♀	13 ♂	50	1.0	Mixed	F	Yes	4	L	No	5	-	1	2	0	1
Loeb 1980 [156]	M, F	+, -	51 ♀	47 ♂	52	10.0	NAC	T	Yes	2	H	No	7	-	1	1	33	1
Longeway 1983 [157]	M	+, -	20 ♀	20 ♂	50	9.0	-	T	Yes	4	L	No	30	-	1	2	0	2
Maccoby et al. 1984 [52]	M	+, -	29 ♀	28 ♂	51	1.3	-	T	Yes	-	Mix	No	17	-	1	2	0	1
Mandara et al. 2012 [38]	M	+, P	55 ♀	44 ♂	56	11.5	AA	T	Yes	4	L	No	10	-	1	2	40	1
Margolin et al. 1975 [158]	M, F	+, -	14 ♀	14 ♂	50	8.4	-	N	Yes	-	H	No	45	2	1	2	50	1
Martinez 1988 [159]	M	+, -, H	28 ♀	19 ♂	60	5.3	SA	T	Yes	1	H	No	10	-	1	2	0	1
McFadyen-Ketchum 1996 [160]	M	-	69 ♀	74 ♂	45	5.0	Mixed	N	Yes, No	4	H	No	120	-	1	1	100	1
McLaughlin et al. 1980 [161]	M, F	-	12 ♀	12 ♂	50	5.0	-	T	Yes	2	L	Yes	23	-	1	1	100	1
McLaughlin 1983 [162]	M, F	-	12 ♀	12 ♂	50	2.5	NAC	F	Yes	2	H	Yes	16	-	1	1	100	1
Michnick et al. 1979 [163]	M, F	+, -	6 ♀	6 ♂	50	1.6	-	F, T, M	Yes	4	L	Yes	20	-	1	2	0	1
Minton et al. 1971 [164]	M	+, -, H	41 ♀	49 ♂	46	2.3	-	N	Yes	4	H	No	300	-	2	2	33	1
Morrell et al. 2003 [165]	M	+, -	28 ♀	31 ♂	47	5.0	-	M	Yes	4	Mix	No	-	-	2	1	50	1
Mullis et al. 1985 [166]	M, F	-	16 ♀	16 ♂	50	9.4	-	T	Yes	2	H	Yes	17	-	1	1	50	1
Neppl et al. 2009 [167]	C	+, -	55 ♀	102 ♂	29	2.3	NAC	T	Yes	2	H	No	5	-	1	2	25	1
O’Brien et al. 1987 [168]	M, F	+, -	10 ♀	10 ♂	50	1.9	NAC	T	Yes	2	L	Yes	12	-	1	2	50	1
Oldershaw et al. 1986 [169]	M	+, -,P,H	20 ♀	20 ♂	50	3.0	-	D	Yes, No	2	L	No	40	-	2	2	33	1
Power 1985 [170]	M, F	+, -, H	12 ♀	12 ♂	50	7–13	NAC	F	Yes	3	L	No	5	-	2	1	100	1
Roberts 1983 [171]	M, F	-	19 ♀	11 ♂	63	4.3	-	N	Yes	4	H	No	-	-	2	1	100	2
Robinson et al. 1981 [172]	M, F	+	16 ♀	26 ♂	38	5.2	-	T	Yes, No	4	L	No	5	3	2	2	0	1
Russell et al. 1996 [173]	C	+, -	28 ♀	29 ♂	49	6.8	A	N	Yes	4	H	No	90	-	1	1	100	1
Scaramella et al. 2008 [174]	M	+, -	20 ♀	20 ♂	50	1.5	Mixed	D	Yes	-	Mix	No	-	-	2	2	20	1
Shaw et al. 1998 [175]	M	-	42 ♀	61 ♂	41	2.0	Mixed	D	Yes	1	L	No	-	-	1	1	50	1
Silverman et al. 1995 [176]	M	+, -, P	15 ♀	18 ♂	45	1.5	Mixed	F, T, M	Yes	4	H	No	12	-	2	1	50	1
Smith et al. 1977 [177]	C	+, -	16 ♀	16 ♂	50	1.5	WEC	N	Yes	4	H	No	60	3	1	1	50	1
Smith et al. 1997 [178]	M	-, H	372 ♀	343 ♂	52	2.0	Mixed	N	No	4	H	No	-	-	1	2	0	1
Smith et al. 2004 [53]	M	-	67 ♀	58 ♂	54	4.5	Mixed	T	No	4	L	No	22	-	1	2	20	1
Smith 2010 [179]	M	-	68 ♀	72 ♂	49	2.7	Mixed	F	Yes	4	L	No	8	-	2	2	0	1
Tam et al. 2003 [180]	M, F	+, -	41 ♀	40 ♂	51	9.8	C	T	Yes	-	L	No	20	-	2	2	0	1
Tamis-LeMonda et al. 2009 [39]	M	+, -	53 ♀	66 ♂	45	6.5	AA	D	Yes	4	-	No	20	-	1	2	50	1
Thomson et al. 2014 [181]	M	+, -	49 ♀	111 ♂	31	1.0	Mixed	T	Yes	4	L	No	4.3	-	2	2	44	1
Trautmann-Villaba et al. 2006 [182]	F	-	45 ♀	43 ♂	51	2.0	WEC	F	Yes	-	L	No	5	-	1	2	67	1
Tulananda et al. 2001 [183]	M, F	+, -, H	31 ♀	22 ♂	58	3.9	Thai	N	Yes	2	H	No	120	2	1	2	50	1
Van Zeijl et al. 2007 [44]	M	+, -	107 ♀	127 ♂	46	2.3	WEC	D	No	4	L	No	10	-	2	2	25	1
Webster-Stratton et al. 1999 [43]	M, F	P	32 ♀	88 ♂	27	5.7	Mixed	N	No	4	H	No	30	-	2	2	0	1
Wilson 1980 [184]	M	+, -	30 ♀	30 ♂	50	3.5–7.5	NAC	T	Yes	3	L	No	10	-	1	2	0	2
Yagmur et al. 2014 [185]	M	+, -	31 ♀	45 ♂	41	2.6	T	D	Yes	-	H	No	9	-	2	2	0	1
Yaman et al. 2010 [186]	M	+, -	58 ♀	82 ♂	41	2.0	WEC, T	D	No	-	H	No	4	-	2	2	20	1
Zevalkink et al. 2001 [187]	M	+, -	36 ♀	40 ♂	47	3.2	In	T	Yes	1	L	No	15	2	2	2	0	1

^a^ M = mother; F = father; C = combined sample.

^b^ + = autonomy-supportive strategy;— = controlling strategy; P = psychological control; H = harsh physical discipline

^c^ AA = African-American; C = Chinese; NAC = North-American Caucasian; SA = South-American; WEC = Western-European Caucasian; I = Israeli; In = Indonesian; A = Australian; T = Turkish.

^d^ D = discipline task; F = free play; N = naturalistic setting; T = teaching/problem-solving task; M = mixed

^e^ SES; 1 = low; 2 = middle; 3 = high; 4 = mixed

^f^ Setting: H = Home; L = Lab

^g^ Other moderators: 1) observation length in minutes; 2) gender of coders (1 = male, 2 = female, 3 = mixed); 3) study goal (1 = examine gender differences, 2 = not examining gender differences), 4) gender first author (1 = male, 2 = female), 5) percentage male authors, 6) publication type (1 = journal, 2 = dissertation).

### Conceptual Analysis: the Sorting Task

Because the grouping of dependent variables may have an important effect on the outcome of a meta-analysis, a sorting task with experts was used (see [19], [188]). Experts were defined as persons who had been trained and actively involved in research on parenting for several years and who were at least participating in a relevant graduate program. A total of 10 experts were asked. Five of the coders had a doctoral degree; the others were advanced graduate students.

Overall, 313 parental control constructs were identified from the selected publications. Because some of the 313 constructs were almost identical, the first, second, and third authors together grouped the constructs that were obviously (near-)identical. Any differences were resolved through discussion and consensus. The grouping resulted in a set of 147 different constructs. Each construct was printed on a separate card, including the definition that was given in the paper and examples of the specific parenting construct. Any information about the source of the construct was left out. Separate sets of cards were made for the four settings in which parental control was observed (e.g., free play, problem solving, discipline setting, naturalistic). This was done because certain aspects of parental control may be evaluated differently depending on the setting in which it was observed [29]. Experts were asked to sort the constructs into three groups of parental control (appropriate/positive, not-appropriate/negative, and neutral, with regard to optimal child development), separately for the four different observation settings. The appropriate/positive and not-appropriate/negative categories correspond with the autonomy-supportive and controlling strategies as proposed by self-determination theory [12]. A neutral category was included only for the sorting task, because we wanted to examine only the most pure forms of controlling and autonomy-supportive strategies in the actual meta-analysis.

Agreement between the experts was satisfactory (kappas .66–.82, average .75). For 117 of the constructs, at least 8 out of 10 experts agreed on sorting the construct in the appropriate/positive, not-appropriate/negative, or neutral control category. The 30 remaining constructs with 70% agreement or less were discussed by the first and last authors. For 12 of these 30 constructs the two authors reviewing the experts’ sorts agreed on one of the existing categories. The remaining 18 constructs were ambiguous or contained both positive and negative elements in one composite score, and therefore could not be grouped under autonomy-supportive or controlling strategies. The outcomes of the expert sort can be found in S2 Table.

Further, the constructs that were identified by the experts as controlling (*n* = 60) were divided in psychological control and harsh physical discipline by the first and second authors. This search was guided by the content of questionnaires and observation scales that are widely used to assess psychological control (i.e., Child Report of Parental Behavior Inventory; [189], Parental Psychological Control measure; [28], Psychological Control Scale; [10]). The psychological control concepts that are assessed with these instruments are: love withdrawal (i.e., parental attention, love, and care is contingent upon children’s compliance with parental requests), erratic emotional behavior (i.e., inconsistent emotional behavior directed at the child), invalidation of the child’s feelings (i.e., tell the child how to feel or think), constraining verbal expressions (i.e., speaking for the child), negative criticism (i.e., shame, disappointment, personal attack), guilt induction (i.e., continually reminding the child of all the sacrifices parents have made to pressurize the child to comply with parents’ requests).

Of the 60 controlling constructs that were examined, only five controlling strategies could be considered indices of psychological control: contingent emotional support (i.e., withdrawal of emotional support after child failure), critiquing/humiliating (i.e., expressing disappointment or criticizing when the child fails to meet expectations), parental negativity (i.e., critical or hostile comments, negative commands, sarcastic and condescending remarks), negatives/negativity (i.e., cold, neglect, reprimands, criticism, corrections), and criticism/critical statements. Five constructs were considered indices of harsh physical discipline: harsh physical discipline, physical power, negative physical control, physical punishment, physical force. The remaining constructs contained a mix of physical, psychological and verbal control (e.g., [81], [145], [146], [175]) or were not defined specifically enough (e.g.,[129]; harsh-intrusive parenting), and were therefore not included in the meta-analyses on psychological control and physical discipline.

### Data Extraction

Three types of moderators were coded: sample characteristics, procedural moderators, and publication moderators (S3 Table). Sample characteristics included the child’s age at the time of the assessment (continuous and categorical; 0–2 years, 2–4 years, 4–18 years), the percentage of girls in the sample (continuous), the socioeconomic background (high, middle, low, mixed), the ethnicity of the sample (African-American, Chinese, North-American Caucasian, West-European Caucasian, South-American, mixed), and the clinical/at-risk status of the sample. Regarding the ethnicity of the sample, samples that were heterogeneous in terms of ethnicity were coded as mixed. Ethnicities other than the ones mentioned above were too uncommon to form a separate category for moderator analyses (i.e., one Australian sample, one Turkish sample, one Indonesian sample, two Israeli samples, one Thai sample). The sample was considered clinical/at risk if the child’s score on a clinical instrument was in the clinical range, if a clinical diagnosis was established, or when a sub-sample of a normal sample with highest/lowest scores on a clinical screening instrument was distinguished. Sample size was also coded, in order to assign weight to the effect sizes. Outcomes were included in the form of, in hierarchical order: (a) mean and standard deviation for parental use of control in boys and girls; (b) correlations between child gender and parental control; (c) *p*-values; (d) statements that there were no differences.

Procedural moderators regarding the measurement of parental control were the setting of the observation (home or laboratory), the observation context (free play, problem solving, discipline task, or naturalistic), the observation length (continuous and categorical; 0–10 minutes, 10–60 minutes, more than 60 minutes), whether the behavior observed was mainly verbal or a mix of verbal and nonverbal behaviors (verbal, mixed), the coders’ gender (100% male, 100% female, mixed), and whether the frequency of parental control behaviors was controlled for the frequency of child behaviors (e.g., proportion scores, analysis with child behavior as covariate) or not.

Publication moderators were gender of the first author, percentage of male authors (continuous and categorical; 0–30%, 31–70%, more than 70%), publication outlet (journal, dissertation), and year of publication (continuous and categorical; before 1980, 1981–1990, 1991–2000, after 2000).

To assess intercoder reliability, 30 publications were coded by the first and the second author. Agreement between the coders was satisfactory for both the moderators and outcome variables (kappas for categorical variables between .63 and 1.00, average .86, and agreement between 85% and 100%, average 96%; intraclass correlations for continuous variables between .98 and 1.00, average .996). Coders reached complete agreement in the reliability set on whether or not test statistics were present. Disagreements between the authors were resolved by discussion. After the reliability assessment, the first author coded the remainder of the articles, but consulted one or more of the other authors in cases of doubt.

### Meta-Analytic Procedures

The meta-analyses were performed using the Comprehensive Meta-Analysis (CMA) program [190]. For each study, an effect size (standardized mean difference, *d*) was calculated. In general, when studies reported analyses with and without covariates, statistics from the analysis without covariates were used. Effect sizes indicating a difference between parental control of boys and girls that was in line with our hypotheses (e.g., more controlling with boys than with girls, more autonomy-supportive strategies with girls than with boys) were given a positive sign, differences that were not in line with our hypotheses were given a negative sign. According to Cohen [191], effect sizes of *d* = 0.20 are considered small, *d* = 0.50 is a medium-sized effect, and *d* = 0.80 is a large effect.

#### Statistical analyses

Combined effect sizes were computed in CMA. Significance tests and moderator analyses were performed through random-effect models, which are more conservative than fixed-effect models. In the random-effect model, the true effect could vary between studies, depending on characteristics of the specific sample. Because of these different characteristics, there may be different effect sizes underlying different studies [192]. To test the homogeneity of the overall and specific sets of effect sizes, we computed Q-statistics [192]. In addition, we computed 95% confidence intervals (CIs) around the point estimate of each set of effect sizes. Q-statistics and *p*-values were also computed to assess differences between combined effect sizes for specific subsets of study effect sizes grouped by moderators. Contrasts were only tested when at least two of the subsets consisted of at least four studies each [193]. Different meta-analyses were conducted for autonomy-supportive and controlling strategies, and for mothers and fathers. Differences in (absolute values of) combined effect sizes between mothers and fathers for specific subsets of study effect sizes grouped by moderators were examined by comparing the 85% CIs. Non-overlapping CIs indicate a significant difference [194], [195], [196], [197].

Funnel plots for each subset were examined in order to detect possible publication bias. A funnel plot is a plot of each study’s effect size against its standard error (usually plotted as 1/SE, or precision). It is expected that this plot has the shape of a funnel, because studies with smaller sample sizes (larger standard errors) have increasingly big variation in estimates of their effect size as random variation becomes increasingly influential, representing the broad side of the funnel, whereas studies with larger sample sizes have smaller variation in effect sizes, which represents the narrow end of the funnel [198], [199]. However, smaller studies with non-significant results or with effect sizes in the non-hypothesized direction are less likely to be published, whereas for large studies, publication of small or non-significant effect sizes or effect sizes in the non-hypothesized direction is more likely because large studies are generally deemed more trustworthy. Therefore, a funnel plot may be asymmetrical around its base (i.e., for small studies no effect sizes for non-significant results or results in the non-hypothesized direction). The degree of asymmetry in the funnel plot was examined by estimating the number of studies which have no symmetric counterpart on the other side of the funnel [198], [200].

We checked for outlying effect sizes and sample sizes separately for the different subsets of studies. *Z*-values below 3.29 or greater than 3.29 were considered outliers [201]. Five outlying effect sizes were detected ([117] fathers’ autonomy-supportive strategies; [143] both mothers’ and fathers’ autonomy-supportive and controlling strategies) and seven studies had outlying sample sizes [47], [82], [120], [127], [128], [146], [178]. Analyses were conducted with and without studies with outlying effect sizes. The outliers with regard to sample size were winsorized (highest non-outlying number + difference between highest non-outlying number and before highest non-outlying number).

## Results

### Parents’ Differential Use of Controlling Strategies with Boys and Girls

The combined effect size for the difference in parental controlling of boys and girls was non-significant (*d* = 0.05, 95% CI [-0.01, 0.11], *p* = .08). The set of studies was highly heterogeneous (*Q* = 498.64, *p* < .01). Excluding outlying effect sizes (*k* = 2), the combined effect size was significant but small (*d* = 0.08, 95% CI [0.05, 0.12], *p* < .01; Table 2) in a heterogeneous set of studies (*Q* = 224.94, *p* < .01). The effect size was positive, indicating that parents used more controlling strategies with boys than with girls. Moderator analyses were conducted without outliers.

**Table 2:** Parents’ Controlling Behaviors.

Characteristics	*k*	*N*	*d*	95% CI	*Q*
Total set	161	15,679	0.082**	[0.045, 0.120]	224.94**
*Sample*					
Parent gender					1.59
Father	35	2,633	0.124**	[0.058, 0.190]	30.33
Mother	118	12,238	0.077**	[0.044, 0.109]	173.58**
Mixed	8	808	0.058	[-0.070, 0.186]	19.29**
Child age					6.01*
0–2 years	41	3,525	0.158**	[0.099, 0.217]	28.05
2–4 years	40	5,104	0.037	[-0.058, 0.132]	97.20**
> 4 years	80	7,050	0.081**	[0.035, 0.127]	89.67
Normative sample					4.33*
Yes	140	12,181	0.111**	[0.078, 0.145]	142.63
No	21	3,498	-0.029	[-0.156, 0.099]	69.84**
SES					1.86
Low	16	1,323	0.073	[-0.061, 0.207]	23.22
Middle	27	2,841	0.119**	[0.049, 0.190]	27.02
High	26	1,232	0.029	[-0.083, 0.142]	5.42
Mixed	72	9,220	0.086**	[0.025, 0.146]	153.72**
Ethnicity					5.61
African-American	4	529	0.278**	[0.050, 0.506]	4.49
N-A Caucasian	28	1,461	0.085	[-0.014, 0.184]	7.65
Chinese	5	452	0.127	[-0.033, 0.286]	2.08
W-E Caucasian	7	1,002	0.185	[-0.028, 0.398]	14.36*
South-American	5	224	0.162	[-0.210, 0.534]	9.28
*Procedure*					
Verbal					0.64
Only	17	458	0.001	[-0.203, 0.204]	18.50
Mixed	144	15,221	0.085**	[0.047, 0.123]	205.87**
Setting					0.51
Home	67	7,652	0.098**	[0.058, 0.138]	58.70
Lab	88	7,561	0.071*	[0.009, 0.133]	153.89**
Task					2.82
Free play	30	2,887	0.089	[-0.054, 0.233]	78.00**
Naturalistic	33	3,164	0.111**	[0.054, 0.168]	23.29
Teaching	69	7,019	0.057*	[0.007, 0.108]	77.71
Discipline	25	2,515	0.136*	[0.028, 0.243]	38.03*
Observation length					1.91
0–10 minutes	52	5,704	0.036	[-0.049, 0.121]	103.14**
11–60 minutes	76	7,336	0.099**	[0.052, 0.146]	86.05
> 60 minutes	15	922	0.113*	[0.013, 0.213]	7.64
Coders gender					0.01
Female	13	981	0.023	[-0.084, 0.130]	9.05
Mixed	4	199	-0.008	[-0.524, 0.507]	8.93*
Control child behavior					1.17
Yes	14	1,000	0.156*	[0.030, 0.283]	17.78
No	99	7,794	0.083**	[0.042, 0.125]	85.21
*Publication*					
Gender first author					1.29
Male	53	3,797	0.049	[-0.010, 0.108]	52.88
Female	108	11,882	0.093**	[0.047, 0.139]	170.16**
% male authors					1.37
0–30	72	7,987	0.064*	[0.004, 0.124]	125.52**
31–70	59	6,227	0.109**	[0.055, 0.163]	71.36
> 70	30	1,465	0.067	[-0.024, 0.158]	29.90
Publication outlet					0.14
Journal	142	14,038	0.084	[0.042, 0.126]	214.38**
Dissertation	19	1,641	0.067	[-0.014, 0.148]	10.35
Publication year					1.66
< 1980	17	757	0.150**	[0.042, 0.257]	19.09
1981–1990	54	2,083	0.098*	[0.021, 0.174]	43.10
1991–2000	33	4,340	0.072*	[0.017, 0.126]	24.47
> 2000	56	8,499	0.088*	[0.018, 0.158]	136.02**

*Note*. Statistics displayed are from analyses without outliers. Abbreviations stand for North-American (N-A), Western-European (W-E), number of samples (*k*), sample size (*N*), standardized mean difference (*d*), 95% confidence interval (CI), heterogeneity (*Q*).

* *p* < .05,

** *p* < .01

The combined effect size for the normative group (*d* = 0.11, 95% CI [0.08, 0.15], *p* < .01, *k* = 140, *n* = 12,181) was larger than the combined effect size for the group with clinical or at-risk samples (*d = -*0.03, 95% CI [-0.16, 0.10], *p* = .66, *k* = 21, *n* = 3,498; *Q*_*contrast*_ (1) = 4.33, *p* < .05), indicating that the differential controlling of boys and girls was larger in normative groups than in clinical and at-risk groups, where the gender difference was absent. Child age was also a significant moderator (*Q*_*contrast*_ (2) = 6.01, *p* < .05), indicating that the combined effect size was largest in the youngest age group (0–2 years; *d* = 0.16, 95% CI [0.10, 0.22], *p* < .01, *k* = 41, *n* = 3,525), followed by the oldest age group (> 4 years; *d* = 0.08, 95% CI [0.04, 0.13], *p* < .01, *k* = 80, *n* = 7,050) and the middle age group (2–4 years; *d* = 0.04, 95% CI [-0.06, 0.13], *p* = .44, *k* = 40, *n* = 5,104). The contrast between the youngest age group and the two older groups was also significant (*Q*_*contrast*_ (1) = 5.86, *p* < .05). None of the other moderators were significant. Continuous moderators were tested using meta-regression analyses, but none of them were significant.

#### Differences between mothers’ and fathers’ gender-differentiated use of controlling strategies

To test whether mothers’ and fathers’ differential controlling of boys and girls was dependent on different moderators, two meta-analyses were conducted, separately for mothers and fathers. The combined effect size for mothers’ differential controlling of boys and girls was small but significant (*d* = 0.07, 95% CI [0.03, 0.12], *p* < .01) in a heterogeneous set of studies (*Q* = 173.58, *p* < .01). The combined effect size for fathers was also significant (*d* = 0.12, 95% CI [0.06, 0.19], *p* < .01) in a heterogeneous set of studies (*Q* = 30.33, *p* < .01). Although the effect size for fathers was slightly higher than that for mothers, the 85% confidence intervals of mothers (85% CI [0.04, 0.11]) and fathers (85% CI [0.08, 0.17]) overlapped, indicating that mothers and fathers did not differ in the extent of their differential treatment of boys and girls; both were more controlling with their boys more than with their girls. For mothers, observation time was a significant moderator (*Q*_*contrast*_ (1) = 3.91, *p* < .05), next to child age and normativity of the sample. Mothers used more controlling strategies with boys than with girls but this effect could only be detected with observation longer than 10 minutes (0–10 minutes: *d* = 0.01, 95% CI [-0.10, 0.11], *p* = .91; > 10 minutes: *d* = 0.12, 95% CI [0.07, 0.16], *p* < .01). All 85% CIs for moderators tested in mothers and fathers were overlapping, indicating no differences between mothers and fathers for the effects of the moderators.

#### Parents’ differential use of psychological and harsh physical control with boys and girls

Separate meta-analyses were conducted for two types of controlling strategies: studies specifically examining psychological control (*k* = 15, *n* = 1,226), and studies examining harsh physical discipline (*k* = 18, *n* = 1,190). The gender difference for psychological control was not significant (*d* = -0.00, 95% CI [-0.12, 0.11], *p* = .98) in a homogeneous set of studies (*Q* = 4.04, *p* = .99). The combined effect size for the difference in harsh physical discipline with boys and girls was not significant either (*d* = 0.10, 95% CI [-0.01, 0.22], *p* = .07) in a homogeneous set of studies (*Q* = 7.38, *p* = .98). With regard to the differences between mothers and fathers in the gender-differentiated use of harsh physical discipline, mothers used more harsh discipline with boys than with girls (*d* = 0.12, 95% CI [0.01, 0.24], *p* < .05, *k* = 14, *n* = 1,190). Parent gender was however not a significant moderator of the gender-differentiated use of harsh physical discipline (*Q*_*contrast*_ (1) = 1.22, *p* = .27). The subsets of studies on psychological control and harsh physical discipline were too small to conduct further moderator analyses.

### Parents’ Differential Use of Autonomy-Supportive Strategies with Boys and Girls

The results of the meta-analysis on differential autonomy-supportive strategies with boys and girls indicated that the gender difference was not significant (*d* = 0.03, 95% CI [-0.00, 0.07], *p* = .06) in a homogeneous set of studies (*Q* = 139.09, *p* = .46). Excluding the outlying effect sizes (*k* = 3) did not change the results (*d* = 0.03, 95% CI [-0.00, 0.07], *p* = .07; Table 3), again, the set of studies was homogeneous (*Q* = 108.10, *p* = .96). Further analyses were conducted without outliers. Although the set of studies was not significantly heterogeneous, the value of the *Q* statistic indicated a moderate to large degree of heterogeneity [202]. We therefore conducted moderator analyses to examine this heterogeneity. None of the sample or procedural moderators were significant.

**Table 3:** Parents’ Autonomy-Supportive Strategies.

Characteristics	*k*	*N*	*d*	95% CI	*Q*
Total set	136	12,182	0.032	[-0.002, 0.066]	108.10
*Sample*					
Parent gender					1.33
Father	29	2,027	0.001	[-0.075, 0.076]	15.75
Mother	98	9,094	0.035	[-0.005, 0.075]	88.22
Mixed	9	1,061	0.081	[-0.040, 0.203]	2.80
Child age					1.48
0–2 years	39	2,675	0.007	[-0.062, 0.075]	11.42
2–4 years	34	4,762	0.060	[-0.006, 0.125]	39.80
> 4 years	63	4,745	0.014	[-0.040, 0.068]	54.43
Normative sample					1.24
Yes	118	9,976	0.032	[-0.005, 0.069]	70.54
No	18	2,206	-0.049	[-0.187, 0.089]	37.54**
SES					1.66
Low	13	1,852	-0.011	[-0.101, 0.079]	5.93
Middle	18	1,804	-0.011	[-0.104, 0.081]	7.28
High	23	1,108	0.023	[-0.095, 0.142]	3.40
Mixed	64	6,403	0.045	[-0.011, 0.100]	78.72
Ethnicity					0.66
N-A Caucasian	22	1,185	0.073	[-0.042, 0.187]	4.00
Chinese	6	452	0.024	[-0.135, 0.182]	0.99
W-E Caucasian	6	758	0.109	[-0.039, 0.256]	5.14
South-American	4	144	0.115	[-0.215, 0.446]	1.07
*Procedure*					
Verbal					0.97
Only	13	449	0.123	[-0.062, 0.309]	2.82
Mixed	123	11,733	0.029	[-0.006, 0.063]	104.32
Setting					1.34
Home	54	4,556	0.006	[-0.049, 0.061]	30.49
Lab	75	6,322	0.049*	[0.002, 0.096]	72.11
Mixed	4	255	0.032	[-0.213, 0.278]	0.25
Task					3.33
Free play	21	1,705	0.092*	[0.002, 0.183]	15.49
Naturalistic	20	1,218	0.009	[-0.097, 0.115]	11.44
Teaching	62	6,136	0.009	[-0.040, 0.059]	45.53
Discipline	27	2,550	0.062	[-0.010, 0.134]	13.57
Observation length					0.26
0–10 minutes	49	4,797	0.039	[-0.017, 0.095]	48.74
11–60 minutes	63	4,895	0.020	[-0.032, 0.071]	51.52
> 60 minutes	10	701	0.032	[-0.107, 0.172]	0.70
Coders gender					0.81
Female	13	981	-0.057	[-0.174, 0.059]	5.79
Mixed	9	536	0.038	[-0.134, 0.210]	0.89
Control child behavior					2.13
Yes	12	708	-0.135	[-0.334, 0.063]	24.57*
No	91	5,702	0.017	[-0.032, 0.065]	42.76
*Publication*					
Gender first author					0.01
Male	42	3,283	0.033	[-0.030, 0.097]	18.77
Female	94	8,899	0.031	[-0.009, 0.071]	89.32
% male authors					0.26
0–30	61	5,725	0.041	[-0.009, 0.091]	45.68
31–70	53	5,291	0.024	[-0.030, 0.077]	52.66
> 70	22	1,166	0.024	[-0.073, 0.120]	9.49
Publication outlet					0.03
Journal	124	11,111	0.031	[-0.005, 0.067]	103.30
Dissertation	12	1,071	0.040	[-0.060, 0.140]	4.77
Publication year					9.00*
< 1980	13	609	-0.004	[-0.145, 0.137]	4.88
1981–1990	44	1,585	-0.076	[-0.162, 0.009]	31.66
1991–2000	30	3,406	0.032	[-0.034, 0.097]	22.22*
> 2000	49	6,582	0.070**	[0.023, 0.117]	40.35

*Note*. Statistics displayed are from analyses without outliers. Abbreviations stand for North-American (N-A), Western-European (W-E), number of samples (*k*), sample size (*N*), standardized mean difference (*d*), 95% confidence interval (CI), heterogeneity (*Q*).

* *p* < .05,

** *p* < .01.

**Fig 2:**
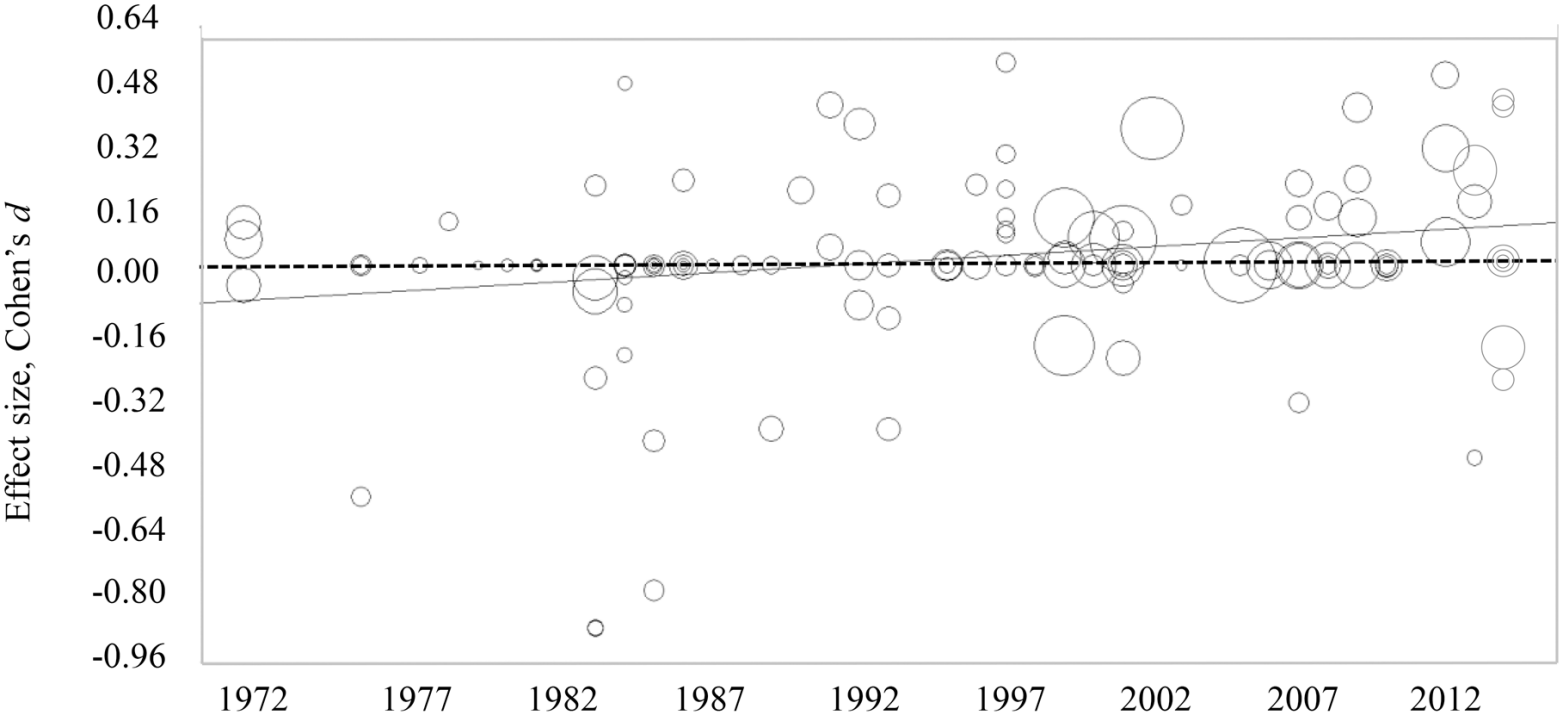
Scatterplot showing the relation between year of publication and Cohen’s d of autonomy-supportive strategies. Note. Solid line represents regression line, dashed line represents Cohen’s d = 0.00.

However, publication year was a significant moderator (*Q*_*contrast*_ (3) = 9.00, *p* < .05), which was confirmed in a meta-regression (*B* = 0.01, 95% CI [0.00, 0.01], *p <* .05). Test of time-related trends showed a significant positive correlation between year of publication (1971–2014) and Cohen’s *d* (*r* = 0.22, *p* = 0.01). Fig 2 displays the relation between year of publication and standardized Cohen’s *d*. In the 70s and 80s, effect sizes are negative, indicating that boys received more autonomy-supportive parenting than girls. From 1990 onward, the positive effect sizes indicate that girls received more autonomy-supportive parenting than boys. Because the scatter plot suggested possible non-linearity in the association between year of publication and Cohen’s *d*, a quadratic function was also tested but this did not fit the data better than the linear function (both models *z* = 2.56). Because publication year was significantly associated with the moderator observation time (*r* = -.18, *p <* .05) and percentage male authors (*r* = -.17, *p <* .05) a multivariate regression analysis was also conducted, but publication year was the only significant moderator (*B* = 0.01, 95% CI [0.00, 0.01], *p <* .01).

#### Differences between mothers’ and fathers’ gender-differentiated use of autonomy-supportive strategies

To test whether mothers’ and fathers’ use differential autonomy-supportive strategies with boys and girls was dependent on different moderators, two meta-analyses were conducted separately for mothers and fathers. The combined effect size for mothers’ differential autonomy-supportive strategies with boys and girls was not significant (*d* = 0.04, 95% CI [-0.01, 0.08], *p* = .09) in a homogeneous set of studies (*Q* = 88.22, *p* = .73). The combined effect size for fathers was also not significant (*d* = 0.00, 95% CI [-0.08, 0.08], *p* = .99) in a homogeneous set of studies (*Q* = 15.75, *p* = .97). For both mothers and fathers, none of the moderators were significant.

### Publication Bias

There was no evidence for publication bias in the funnel plots (see S1 and S2 Figs). Using the trim and fill method [198], [200], asymmetries (missing studies in the non-hypothesized direction) were not found in the meta-analyses on controlling and autonomy-supportive strategies.

## Discussion

Surprisingly few differences were found in parents’ use of control with boys and girls. Of the four different types of observed parental control (including autonomy-supportive strategies, overall controlling strategies, psychological control, and harsh physical control), parents only differentiated between boys and girls with regard to overall controlling strategies. Parents were slightly more controlling with boys than with girls, but the effect size can be considered negligible. Some significant but very small moderator effects were found. First, the combined effect size for controlling strategies was larger for younger children than for older children and larger in normative groups than in at-risk and clinical groups. Second, parents showed more autonomy-supportive strategies with boys than with girls before 1990, whereas in studies from 1990 onward, parents showed more autonomy-supportive strategies with girls than with boys. Contrary to our expectations, mothers and fathers did not differ in the extent to which they used differential parental control with boys and girls.

The nonsignificant and small effect sizes for gender-differentiated parental control imply that there is considerable similarity in parents’ control of boys and girls. As parental control plays an important role in children’s development of autonomous or controlled regulation of behavior [13], [14], parents appear to use similar levels of autonomy-supportive parenting and controlling parenting with boys and girls to support optimal development in both sons and daughters. These findings argue against the propositions of biosocial theory that parents use gender-differentiated parenting as a means of gender-role socialization. Apparently, mothers and fathers do not use different control strategies with boys and with girls to prepare them for their future gender roles in society. It is possible that parents do not regard child outcomes associated with parental control (e.g., self-regulation) as relevant to masculinity or femininity, and therefore do not socialize boys and girls differently with regard to control [203]. Parents might use more specific and subtle gender socialization practices to influence their children’s gender-role behavior. There is evidence that gender differentiation and discrimination has become less blatant and increasingly subtle in many contemporary societies [204]. In addition, larger and more consistent differences in the treatment of boys and girls are found with regard to parents’ encouragement of gender-typical activities [8], parental gender talk [205], and parents’ toy, clothing, and chore choices for children [206]. Moreover, two large longitudinal studies focusing on gender-specific emotion socialization [95] and physical discipline in response to boys’ and girls’ noncompliance [207] have found that fathers differential socialization of boys and girls was related to larger gender differences in child behavior a year later [95]. In the current meta-analysis we did not find evidence for parents using the specific strategies harsh physical control and psychological control differently with boys and girls, which might be due to a lack of power.

Our findings are not necessarily discordant with the argument of gender schema theories [4] that parents’ gender-differentiated use of controlling and autonomy supportive strategies is likely to be influenced by parents’ gender-role stereotypes. In the current meta-analysis we were unable to examine whether parents’ gender stereotypes influenced gender-differentiated parenting practices, as hardly any studies provided pertinent data. Parents with traditional attitudes about gender roles might have been more likely to show gender-differentiated parenting that reinforces gender-role consistent behavior (e.g., more harsh or physical control of boys than girls, more gentle control and guidance of girls than of boys) than other parents.

Some significant moderators of parents’ gender-differentiated use of control were found, but the effect sizes were very small. First, the effect size for controlling strategies was largest in studies with children between 0 and 2-years-old, a time in which gender differences in disruptive behavior or difficult temperament are generally less pronounced [59], [208], [209]. This finding argues against parents’ gender-differentiated use of controlling strategies being elicited by pre-existing gender differences in behavior (i.e., child-elicited effect). Second, the finding that differential controlling of boys and girls was detected in studies that used normative samples rather than clinical or at-risk samples might indicate specific interaction dynamics in families experiencing problems. Third, with regard to autonomy-supportive strategies, we found that in earlier studies parents used more autonomy-supportive strategies with boys than toward girls, whereas from 1990 onward, parents used more autonomy-supportive strategies with girls than toward boys. These findings might be attributable to historic changes in child rearing, with a strong parental preference for and involvement with sons in the 70s and 80s changing to a greater preference for and involvement with daughters after 1990 [210], [211], [212], [213], [214].

The majority of the moderators were not significant. Most importantly, mothers and fathers did not differ in the extent of their differential use of controlling or autonomy-supportive strategies with boys and girls. This was unexpected based on biosocial theory [2], [3] and previous findings of more gendered parenting by fathers than mothers [6], [8]. It is possible that mothers and fathers differ in their gender-differentiated parenting practices only with regard to very specific socialization areas, which were not represented in general measures of parental control. Further, we did not find any moderating effect for the observed task or the observational setting. Apparently, the demand level of the observational setting do not influence gendered patterns of parental control. Last, differential control toward boys and girls was not dependent on the socioeconomic status of the family, the ethnicity of the sample, the gender of the first author, the percentage of male authors, or the publication outlet. Especially the null findings with regard to ethnicity and socioeconomic status of the sample were unexpected in light of biosocial theory [2], [3]. It may be that the relatively small number of studies with homogeneous ethnicities or low-SES parents decreased the power to detect effects of ethnicity and SES on gender-differentiated parenting. However, these results could also indicate that the strictness of the gender roles in a family, which is closely linked to ethnicity and SES, are not related to the level of gender-differentiated discipline.

### Limitations and Future Directions

Despite the strengths of the present meta-analytic study, some limitations need to be addressed. First, although we identified several significant moderators of differential control toward boys and girls, there was still considerable variation in effect sizes in some sets of studies. This points to other factors, such as the strength of parents’ gender stereotypes, which may account for variations in gender-differentiated parenting. Lumping together parents with traditional and counter-stereotypical gender attitudes in empirical studies and in the current meta-analysis may have obscured any systematic differences in the differential control of boys and girls. This would also contribute to large differences between studies and individual differences within studies. Future research on gender-differentiated parenting should take parents’ gender stereotypes into account, to further elucidate why some parents do use different parenting strategies with boys and girls and others do not. These studies should also longitudinally investigate the consequences of gender-differentiated parenting for gender differences in child behavior, as very few studies have actually examined parents’ role in the development of gender differences in children’s behavior [38], [39], [95]. Second, the sorting of the parental control constructs was necessary because of conceptual problems with the control construct (i.e., very dependent on the situation), but it has the disadvantage of losing information with regard to behaviors that were grouped under the neutral control category.

Third, it is important to note that almost all studies in this meta-analysis adopted a between-family design to examine differences in parenting boys and girls. This is an approach where parental control in families with boys is compared with the control practices in families with girls. An important limitation of this approach is that differences between boys and girls in parenting practices do not necessarily reflect a gender difference, but can also be caused by other underlying differences in family characteristics, such as family-interaction patterns. It is of vital importance to examine gender-differentiated parenting within families to account for such factors. In the current meta-analysis it was not possible to compare studies that used a between-family design with studies that employed a within-family design, simply because there were too few studies with within-family comparisons. More studies with a within-family design are needed to disentangle the effect of child gender on parenting practices from between-family effects. Such studies also enable testing whether gender-differentiated socialization is more pronounced in families that include both boys and girls compared to families with all girls or all boys [215].

Last, very few observation studies included a focus on harsh physical discipline or psychological control. In most studies the controlling strategies included a mix of physical, psychological, or negative verbal strategies. More studies with a focus on observed psychological control or harsh physical discipline are needed to examine whether parents use these excessive control strategies differently with boys and girls (as opposed to milder controlling strategies). This is especially important because psychological control and harsh physical discipline might be prone to social desirability in self-report studies [216], and because of their detrimental effects on child development [10], [15], [26], [27], [28], [30], [31]. Although psychological control and harsh discipline are difficult to observe in short observation periods, previous research has shown that it can be done reliably and with meaningful results (see [10], [217]). Relatedly, conducting a meta-analysis on studies using questionnaires to assess parental control might have resulted in different findings than the current meta-analysis. Questionnaires can assess a broad range of naturalistic behaviors but have the disadvantage of reporter bias, whereas observations, albeit more objective, focus on specific behaviors in a structured setting with an experimenter present. However, the literature on (self-) reported gender-differentiated parental control is as inconsistent as the literature on observed parental control. Some studies found no differences between boys and girls (e.g., [75], [218]), others showed that girls received more autonomy support (e.g., [219]) or controlling parenting (e.g., [220]) than boys, or that boys received more autonomy support (e.g., [221]) or controlling (e.g., [222]) than girls.

### Conclusion

The current meta-analytic study extends previous meta-analytic work from the 1990s on parents’ differential behavior toward boys and girls by focusing on observations of verbal and physical parental control in a variety of settings and contexts, and by providing a contemporary update. Overall, the effects of child gender on parents’ use of control were very small, indicating large similarities in parents’ control strategies with boys and girls. These findings question the importance of gender-differentiated parental control as a means of gender socialization and as a mechanism underlying gender differences in child behavior. However, the large differences between studies and the individual differences within studies suggest that some parents do treat their sons and daughters differently with regard to parental control. Parents’ gender stereotypes might explain why some parents do treat their sons and daughters differently and others do not, but this mechanism has yet to be confirmed empirically.

## Supporting Information

S1 Fig Funnel plot for meta-analysis on controlling strategies. (TIF)

S2 Fig Funnel plot for meta-analysis on autonomy-supportive strategies. (TIF)

S1 Table Additional Restrictions in the Literature Search in Web of Science. (DOCX)

S2 Table Outcomes of Expert Sort for Parental Control Constructs. 1. Less than 80% agreement, consensus through discussion. 2. Contains positive and negative elements or composite score. 3. Dependent on tone of voice and/or situation.4. Too few information to judge.
(DOCX)

S3 Table Coding System for Meta-Analysis. (DOCX)

S1 Text PRISMA checklist. (DOC)

S2 Text Search strategy. (DOCX)
