# Supplementary figures and images for: Gender-Differentiated Parenting Revisited: Meta-Analysis Reveals Very Few Differences in Parental Control of Boys and Girls
Source: PLoS One. 2016 Jul 14;11(7):e0159193. doi: 10.1371/journal.pone.0159193 (PMC4945059; doi:10.1371/journal.pone.0159193)

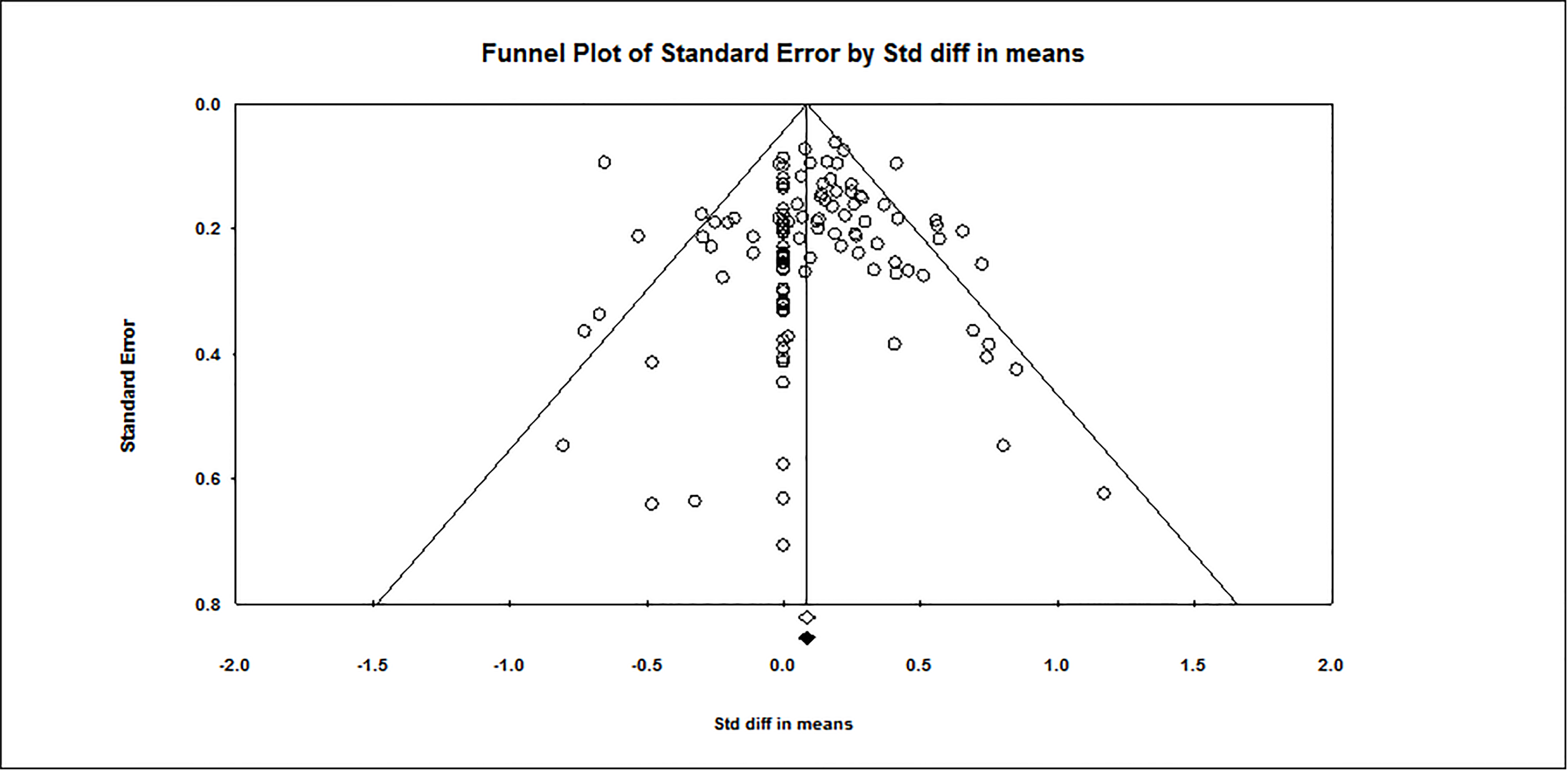

Supplement: S1 Fig — (TIF) [file pone.0159193.s001.tif]

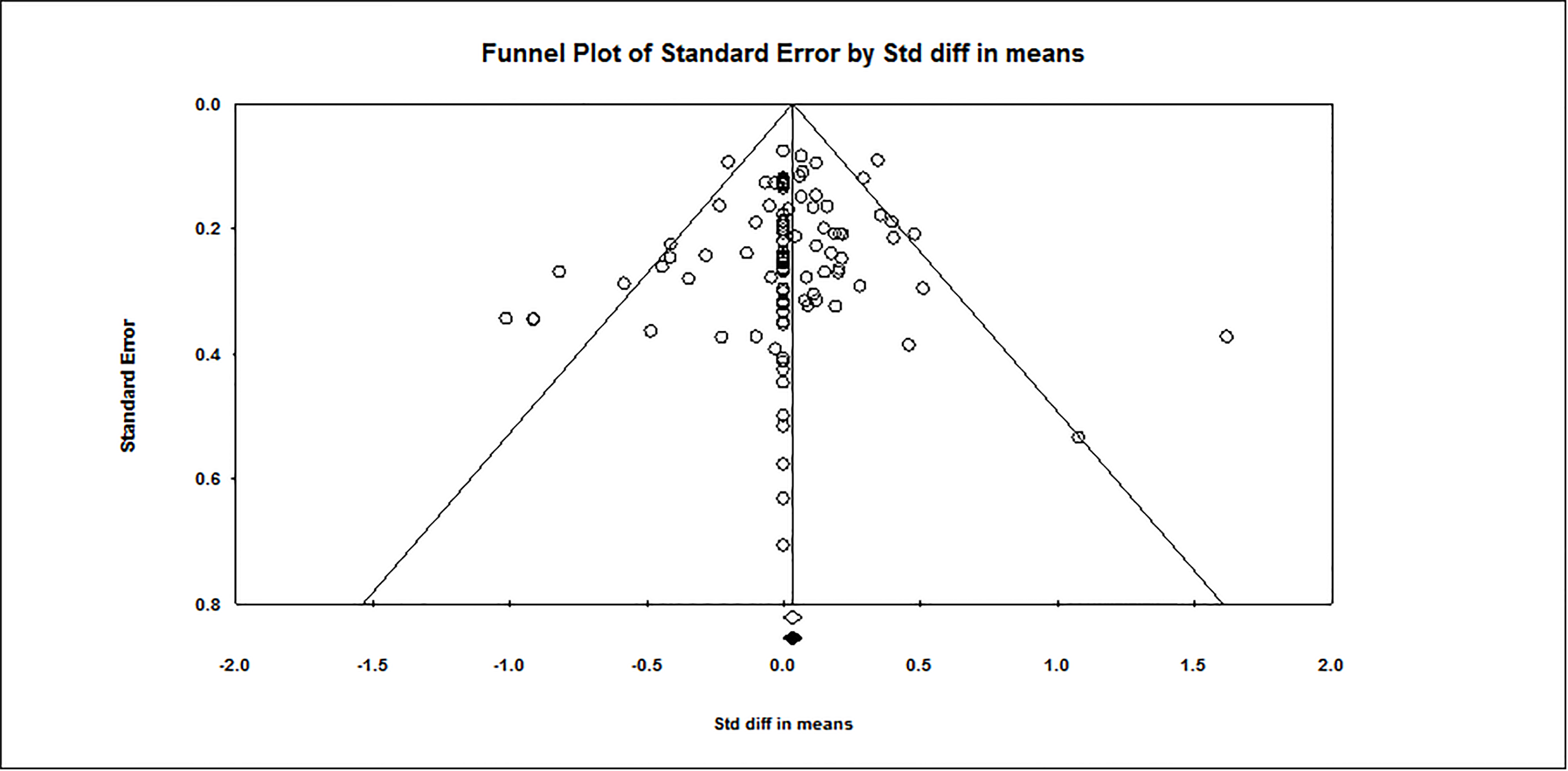

Supplement: S2 Fig — (TIF) [file pone.0159193.s002.tif]
