# Supplementary material for: Gender-Differentiated Parenting Revisited: Meta-Analysis Reveals Very Few Differences in Parental Control of Boys and Girls
Source: PLoS One. 2016 Jul 14;11(7):e0159193. doi: 10.1371/journal.pone.0159193 (PMC4945059; doi:10.1371/journal.pone.0159193)
Supplement: S1 Table — (DOCX) [file pone.0159193.s003.docx]

**S1 Table**. Additional restrictions in the literature search in Web of Science.

The following Web of Science categories were excluded from the search, because original search yielded too many unwanted hits, mostly medical (3,257 as opposed to 1,676 with categories excluded).

| Public environmental occupational health | Cell biology |
| --- | --- |
| Nutrition dietics | Dentistry |
| Obstetrics gynecology | Surgery |
| Medicine general internal | Ethics |
| Immunology | Veterinary sciences |
| Anesthesiology | Biophysics |
| Endocrinology metabolism | Demography |
| Nursing | Food science technology |
| Toxicology | Biotechnology applied microbiology |
| Pharmacology pharmacy | Medical ethics |
| Infectious diseases | Medical informatics |
| Health care science services | Parasitology |
| Environmental sciences | Cardiac cardiovascular systems |
| Oncology | Ecology |
| Health policy services | Orthopedics |
| Medicine research experimental | Computer science information systems |
| Computer science artificial intelligence | Medical laboratory technology |
| Allergy | Pathology |
| Respiratory systems | Sport sciences |
| Reproductive biology | Agriculture dairy animal science |
| Biochemistry molecular biology | Computer science interdisciplinary |
| Critical care medicine | Computer science theory methods |
| Tropical medicine | History philosophy of science |
| Hematology | Transplantation |
| Virology | Emergency medicine |
| Zoology | Physiology |
| Microbiology | Radiology |
| Biology | Audiology speech language pathology |
| Gastroenterology hepatology | Otorhinolaryngology |
| Dermatology | Criminology penology |
| Economics | Ophthalmology |
| Urology nephrology |  |
