# Supplementary material for: Gender-Differentiated Parenting Revisited: Meta-Analysis Reveals Very Few Differences in Parental Control of Boys and Girls
Source: PLoS One. 2016 Jul 14;11(7):e0159193. doi: 10.1371/journal.pone.0159193 (PMC4945059; doi:10.1371/journal.pone.0159193)
Supplement: S2 Table — 1. Less than 80% agreement, consensus through discussion. 2. Contains positive and negative elements or composite score. 3. Dependent on tone of voice and/or situation.4. Too few information to judge. (DOCX) [file pone.0159193.s004.docx]

**S2 Table**. Outcomes of expert sort for parental control constructs

| Setting | Autonomy-supportive control | Neutral/appropriate control | Controlling strategies | Ambiguous |
| --- | --- | --- | --- | --- |
| Discipline task | Supportive/positive strategy | Directive | Non-supportive strategy | Physical control strategies^2^ |
|  | Guidance | Neutral command/ prohibitive | Negative control | Encourage appropriate behavior^3^ |
|  | Positive command/ prohibitive | Commands | Critiquing | Control^2^ |
|  | Praise | Verbal control strategies | Power assertion | Control attempts^2^ |
|  | Guiding towards competence | Control | Inappropriate, interfering control | Corrections^3^ |
|  | Warm control discipline | Prohibition | Permissive behavior | Moderate power assertion^2^ |
|  | Developmentally appropriate responses | Neutral redirection^1^ | Physical power | Control/bribe^2^ |
|  | Positive strategies |  | Negative discipline |  |
|  | Authoritative control |  | Negativity |  |
|  |  |  | Rejecting |  |
|  |  |  | Negative strategies |  |
|  |  |  | Intrusive, negative verbal feedback |  |
|  |  |  | Authoritarian control |  |
|  |  |  |  |  |
| Free play | Affectionate terms | Assertions | Harsh vocalization | Directing^2^ |
|  | Explain/reason/ suggest |  | Control/direct/command |  |
|  | Positive vocalization |  | Negative control |  |
|  | Praise/positives |  | High power strategies |  |
|  | Positive comments |  | Harsh-intrusive parenting |  |
|  | Gentle guidance |  | Authoritarian parenting |  |
|  | Positive responses |  | Physically force |  |
|  | Assist/demonstrate^1^ |  | Demanding |  |
|  | Low power strategies^1^ |  | Restrictive |  |
|  | Authoritative parenting^1^ |  |  |  |
|  |  |  |  |  |
| Naturalistic | Encourage independence | Instrumental direction | Firm enforcement | Physical interventions^3^ |
|  | Cooperative instruction | Moving object from child | Rejecting | Instruct/direct^2^ |
|  | Supportive guidance | or child from situation | Authoritarianism | Passive-acceptant^2^ |
|  | Positive parenting | Commands | Beta commands | Demandingness^2^ |
|  | Help/explain | Control | Restrictive |  |
|  | Distraction |  | Coercive |  |
|  | Reasoning^1^ |  | Criticism |  |
|  |  |  | Physical punishment |  |
|  |  |  | Aversive/negative parenting |  |
|  |  |  | Severe discipline/punishment^1^ |  |
|  |  |  |  |  |
| Problem solving/ | Praise/positives | Control (child off-task) | Direct/command | Direct/command^2^ |
| Teaching task | Positive response | Verbal controls | Negative response | Physical manipulation^2^ |
|  | Effective discipline | Control | Controlling/intrusive behavior | Directive teaching^2^ |
|  | Positive/supportive parenting | Controlling behavior^1^ | Negative control/ power assertion | Contingent control^3^ |
|  | Positive interaction | Implicit/indirect control^1^ | Harsh discipline | No control^3^ |
|  | Consistent discipline |  | Inconsistent discipline | Positive physical control^4^ |
|  | Induction |  | Demandingness |  |
|  | Guidance |  | Aversiveness |  |
|  | Positive control |  | Extreme control |  |
|  | Support |  | Negative control |  |
|  | Warmth |  | Authoritarian control |  |
|  | Cognitive assistance |  | Parent makes demands |  |
|  | Positive feedback/reinforcement |  | Parent manipulates |  |
|  | Positive engagement |  | Restrictive control |  |
|  | Authoritative parenting |  | Behavioral control |  |
|  | Authoritative control |  | Intrusive control |  |
|  | Verbal distraction |  | Power assertion |  |
|  | Positive behaviors |  | Negativity |  |
|  | Reasoning |  | Punishment |  |
|  | Suggest |  | Negative teaching |  |
|  | Empathy |  | Controlling/punitive |  |
|  | Encouragement |  | Disapproval |  |
|  | Approval |  | Hostility/angry coercion |  |
|  | Supportive presence |  | Negative physical control |  |
|  | Rewards^1^ |  | Negative feedback |  |
|  | Structure/limit setting^1^ |  | Negative verbal feedback^1^ |  |
|  |  |  | Direct command^1^ |  |
|  |  |  | Contingent withdrawal of emotional support |  |

1. Less than 80% agreement, consensus through discussion
2. Contains positive and negative elements or composite score
3. Dependent on tone of voice and/or situation
4. Too few information to judge
