# Supplementary material for: Gender-Differentiated Parenting Revisited: Meta-Analysis Reveals Very Few Differences in Parental Control of Boys and Girls
Source: PLoS One. 2016 Jul 14;11(7):e0159193. doi: 10.1371/journal.pone.0159193 (PMC4945059; doi:10.1371/journal.pone.0159193)
Supplement: S3 Table — (DOCX) [file pone.0159193.s005.docx]

**S3 Table**. Coding system for meta-analysis

| Variable | Coding |  |
| --- | --- | --- |
| *Sample* |  |  |
| Child age (at time of assessment) | 1 0-2 years | Also continuous |
|  | 2 2-4 years |  |
|  | 3 older than 4 years |  |
| Normative sample | 1 yes |  |
|  | 2 no (at risk or clinical) |  |
| Socioeconomic background | 1 high |  |
|  | 2 middle |  |
|  | 3 low |  |
|  | 4 mixed |  |
| Ethnicity | 1 African-American |  |
|  | 2 Chinese |  |
|  | 3 North-American Caucasian |  |
|  | 4 South-American |  |
|  | 5 Western-European Caucasian |  |
|  | 6 mixed |  |
| Percentage girls |  | Continuous |
| *Procedure* |  |  |
| Type of parental control | 1 positive | Determined by experts |
|  | 2 neutral |  |
|  | 3 negative |  |
| Behavior observed | 1 verbal |  |
|  | 2 mixed |  |
| Setting | 1 home |  |
|  | 2 lab |  |
|  | 3 mixed |  |
| Task | 1 free play |  |
|  | 2 discipline task | Don’t touch, clean-up, delay of gratification |
|  | 3 problem solving |  |
|  | 4 naturalistic |  |
| Observation length | 1 0-10 minutes | Also continuous |
|  | 2 11-60 minutes |  |
|  | 3 more than 60 minutes |  |
| Coders gender | 1 male |  |
|  | 2 female |  |
|  | 3 mixed |  |
| Study goal | 1 examine gender differences |  |
|  | 2 not examining gender differences |  |
| Control for child behavior | 1 yes |  |
|  | 2 no |  |
| *Publication* |  |  |
| Gender first author | 1 male |  |
|  | 2 female |  |
| Percentage male authors | 1 0-30% | Also continuous |
|  | 2 31-70% |  |
|  | 3 more than 70% |  |
| Publication outlet | 1 journal |  |
|  | 2 dissertation |  |
| Year of publication | 1 until 1980 | Also continuous |
|  | 2 1981-1990 |  |
|  | 3 1991-2000 |  |
|  | 4 after 2000 |  |
