# Supplementary material for: Gender-Differentiated Parenting Revisited: Meta-Analysis Reveals Very Few Differences in Parental Control of Boys and Girls
Source: PLoS One. 2016 Jul 14;11(7):e0159193. doi: 10.1371/journal.pone.0159193 (PMC4945059; doi:10.1371/journal.pone.0159193)
Supplement: S2 Text — (DOCX) [file pone.0159193.s007.docx]

# S2 Text. Search strategy

(parent* OR mother* OR maternal OR father* OR paternal) AND (disciplin* OR induct* OR harsh disciplin* OR harsh parent* OR spank* OR authorit* OR obedien* OR disobedien* OR parental control* OR maternal control* OR paternal control* OR complian* OR noncomplian* OR negative interact* OR coerc* OR negative reinforce*, positive reinforce* OR punish* OR prohib* OR forbid* OR critic* OR limit setting OR praise OR guid* OR psychological control* OR behavioral control*) AND (child* OR preschool* OR toddler OR infan* OR adolescen*) AND (observ* OR experiment*)
